# Associations between synovitis and radiographic osteoarthritis progression in the first carpometacarpal and interphalangeal joints: results from the Nor-Hand study

**DOI:** 10.1136/rmdopen-2026-007074

**Published:** 2026-08-20

**Authors:** Lovisa Klingenberg, Ida K Haugen, Hilde Berner Hammer, Øystein Maugesten, Alexander Mathiessen, Marthe Gløersen

**Affiliations:** 1 Center for Treatment of Rheumatic and Musculoskeletal Diseases (REMEDY), Diakonhjemmet Hospital, Oslo, Norway; 2 Department of Circulation and Medical Imaging, Norwegian University of Science and Technology, Trondheim, Norway; 3 Faculty of Medicine, University of Oslo, Oslo, Norway

**Keywords:** Osteoarthritis, Ultrasonography, Magnetic Resonance Imaging

## Abstract

**Objectives:**

To determine whether baseline synovitis detected by ultrasound or MRI predicts radiographic osteoarthritis (OA) progression in the first carpometacarpal (CMC1) and interphalangeal joints.

**Methods:**

We included 201 participants with hand OA from the Nor-Hand cohort. The CMC1 and interphalangeal joints were examined by ultrasound (both hands) and MRI (dominant hand) at baseline. Grey-scale synovitis, power Doppler activity and MRI-defined synovitis were scored on 0–3 scales. Bilateral hand radiographs at baseline and follow-up were scored by the same reader. We examined whether synovitis at baseline predicted radiographic progression in the same joint defined as ≥1 increase in Kellgren-Lawrence grade, osteophytes or joint space narrowing or incident erosions. CMC1 and interphalangeal joints were analysed separately using logistic regression with generalised estimating equations, adjusted for age, sex, body mass index and follow-up time.

**Results:**

Radiographic Kellgren-Lawrence progression occurred in 14.7% of CMC1 joints and 10.2% of interphalangeal joints. Baseline grey-scale synovitis was associated with radiographic progression in the CMC1 and interphalangeal joints with the strongest observed association between grade 3 synovitis and Kellgren-Lawrence progression (OR (95% CI) of 9.3 (2.2 to 39.6) and 23.4 (10.4 to 52.9), respectively). Similar relationships were found for power Doppler activity and MRI-defined synovitis.

**Conclusions:**

Baseline synovitis detected by ultrasound and MRI was associated with radiographic progression in both CMC1 and interphalangeal joints, supporting inflammation as a relevant prognostic feature across hand OA phenotypes.

**Trial registration number:**

NCT03083548.

**WHAT IS ALREADY KNOWN ON THIS TOPIC**

- Synovitis is associated with radiographic progression in hand osteoarthritis (OA), mainly shown in interphalangeal joints.
- No previous studies have evaluated its predictive value in the first carpometacarpal (CMC1) and interphalangeal joints separately.

**WHAT THIS STUDY ADDS**

- Our study explores whether synovitis assessed by ultrasound and MRI predicts radiographic progression in the CMC1 and interphalangeal joints separately.

**HOW THIS STUDY MIGHT AFFECT RESEARCH, PRACTICE OR POLICY**

- Our results suggest that synovitis is a relevant treatment target in both CMC1 and interphalangeal joints in patients with hand OA.
- Future studies should explore whether treating synovitis in these joints leads to less radiographic progression.

## Introduction

### Background

 Osteoarthritis (OA) is the most common joint disease worldwide and a leading cause of pain, disability and reduced quality of life, with prevalence increasing markedly with age.^1^ Hand joints are frequently affected, and hand OA is associated with substantially impaired grip strength, fine motor skills, activities of daily living and work ability.^2–4^

Structural joint damage in hand OA is characterised by osteophyte formation, joint space narrowing (JSN), erosions, subchondral cysts, sclerosis and malalignment,^2^ and structural progression has been associated with increased joint tenderness.^5^ Radiographic damage is a core outcome domain in hand OA studies according to the Outcome Measures in Rheumatology (OMERACT) hand OA working group.^6^ Radiographic progression therefore represents a key outcome in observational studies and clinical trials in hand OA.

Synovitis is a well-established feature of hand OA and has been associated with both pain and structural disease progression.^7–11^ Ultrasound-detected synovitis has been shown to be associated with pain and reduced physical function in hand OA,^10^ and longitudinal imaging studies have demonstrated that joints with synovitis progress more rapidly than joints without synovitis.^7–9 11^

The 2023 European Alliance of Associations for Rheumatology (EULAR) classification criteria for hand OA introduce first carpometacarpal (CMC1) OA and interphalangeal OA as separate phenotypes.^12^ Most previous studies have treated hand OA as a single entity without differentiating between joint groups.^7–9^ Currently, it remains unknown whether synovitis has similar prognostic significance in the CMC1 and interphalangeal joints. We therefore aimed to determine whether baseline synovitis detected by ultrasound or MRI predicts radiographic progression in the same joint, examining the CMC1 and interphalangeal joints separately.

## Materials and methods

### Study design and setting

The Nor-Hand study is a hospital-based prospective longitudinal observational cohort conducted at Diakonhjemmet Hospital, Oslo, Norway.^13 14^ Participants were recruited from the rheumatology outpatient clinic and from a multidisciplinary OA education programme. A baseline examination was performed in 2016–2017, and one follow-up visit was conducted in 2019–2021.^13 14^

### Participants

Eligible participants were men and women aged 40–70 years with clinical and/or ultrasound-verified hand OA. Individuals with systemic inflammatory joint diseases (eg, rheumatoid arthritis, psoriatic arthritis and spondyloarthritis), psoriasis or haemochromatosis were excluded. At follow-up, participants were re-screened for development of inflammatory arthritis or psoriasis and were excluded if such conditions had emerged, as these conditions could cause synovitis and structural joint damage through mechanisms distinct from hand OA. Detailed inclusion and exclusion criteria have been published previously in the study protocols.^13 14^ In total, 300 participants were examined at baseline and 213 attended the follow-up visit.^15^ Of those, 201 had available hand radiographs at follow-up.

The 2023 EULAR classification criteria for CMC1 OA and interphalangeal OA were applied using a reduced cut-off value (7 of 13 points based on age, osteophytes, JSN and symptom-structure concordance), as suggested by the expert panel when morning stiffness is not recorded.^12^ To fulfil the entry criterion, we used the first question in the American College of Rheumatology criteria (‘hand pain, aching or stiffness on most days the previous month’) and required pain in at least one target joint (CMC1 or interphalangeal joints) on a hand diagram.

### Ultrasound

Ultrasound was performed at baseline by a trained medical student under supervision from experienced ultrasonographers using a GE Logic S8 machine with fixed settings to ensure standardisation. Bilateral CMC1, first interphalangeal (IP1) and second to fifth proximal and distal interphalangeal (PIP/DIP) joints were systematically scanned in longitudinal planes from radial to ulnar dorsal positions, with transverse views added when required. Grey-scale synovitis and power Doppler activity were scored semi-quantitatively (0–3 scales) according to the validated OMERACT-based scoring system and corresponding reference atlases.^16^ A reliability exercise where the medical student and the experienced ultrasonographer (AM) examined and scored 10 patients independently demonstrated good inter-reader agreement.^10^

### Magnetic resonance imaging

MRI of the dominant hand was performed at baseline using a Siemens Aera 1.5 T scanner with a 16-channel hand/wrist coil, with field of view covering both CMC1 and interphalangeal joints. Contrast-enhanced sequences (Dotarem 0.2 mL/kg body weight) were obtained. Standardised multiplanar acquisitions (T1-weighted, proton density (PD), PD-fat saturated, Volumetric Interpolated Breath-hold Examination (VIBE) sequences) were used. Images were scored for synovitis on 0–3 scales in CMC1 joints using the Thumb base OA MRI scoring system (TOMS)^17^ and in the interphalangeal joints using the Hand OA MRI scoring system.^18^ The images were scored by a trained reader (ØM) after demonstrating good to very good inter-reader reliability with an experienced reader (IKH).^19 20^

### Radiography

Standardised bilateral posterior-anterior hand radiographs were obtained at baseline and follow-up. A trained reader (IKH) scored all CMC1, IP1, PIP and DIP joints for structural features using validated instruments: Kellgren-Lawrence (KL) scoring system (0–4 scales),^21^ Osteoarthritis Research Society International atlas (osteophytes and JSN scored on 0–3 scales)^22^ and the Verbruggen-Veys anatomical phase scoring system (joints in E and R phases were defined as erosive).^23^ Paired baseline and follow-up images were read together in chronological order by the same reader, who was blinded to clinical information and other imaging modalities, but not to the chronological order of the images.^24^

### Statistical analysis

All analyses were conducted using Stata/IC V.18 (StataCorp, College Station, Texas, USA). Baseline characteristics were summarised using appropriate descriptive statistics, including means and SD for normally distributed variables, medians and IQRs for skewed variables and frequencies with percentages for categorical variables.

To assess potential selection bias, we compared baseline characteristics of participants with available follow-up radiographs (n=201) with participants without available follow-up radiographic data (n=99, including 87 participants lost to follow-up and 12 participants without follow-up hand radiographs). Mann-Whitney U test was used for continuous variables, and Pearson’s χ² test was used for categorical variables. Missing imaging data were quantified separately for each imaging modality at baseline (ultrasound, MRI and conventional radiography). We investigated whether ultrasound-detected or MRI-detected synovitis at baseline (independent variables) predicted radiographic progression in the same joint at follow-up (dependent variables), with analyses performed separately for CMC1 and interphalangeal joints. Radiographic progression was defined in four ways: an increase in KL grade ≥1, progression of osteophytes (increase ≥1 grade), progression of JSN (increase ≥1 grade) and incident erosions. KL grade progression was considered a global radiographic progression outcome, while osteophyte progression, JSN progression and incident erosions were included as complementary structural outcomes. For each radiographic outcome, joints that had already reached the maximum score for that outcome at baseline were excluded from the corresponding progression analyses (KL grade=4, JSN=3, osteophyte=3). For analyses of incident erosions, only joints without erosions at baseline were included. Joints missing due to amputation or surgery (eg, trapeziectomy) at baseline or during follow-up were also excluded because radiographic progression could not be evaluated in these joints.

We used logistic regression with generalised estimating equations and an exchangeable correlation matrix to account for within-person correlations between joints. Joints without synovitis served as reference. Results were presented as ORs with 95% CIs. Analyses were adjusted for age, sex, body mass index (BMI) and follow-up time. Intrareader agreement was assessed using weighted kappa coefficients for KL grade, osteophytes and JSN, and kappa coefficients for erosions.

### Ethics

The study is registered at ClinicalTrials.gov (identifier NCT03083548).

## Results

### Baseline characteristics

Baseline clinical and imaging characteristics are presented in tables 1 and 2. A total of 201 participants had available hand radiographs at follow-up and were included in the analyses. When comparing these (n=201) with those lost to follow-up and/or without radiographs at follow-up (n=99), no statistically significant differences were observed in age, sex distribution, baseline KL sum score or baseline ultrasound-detected synovitis sum score (data not shown).

**Table 1:** Baseline clinical characteristics of the study population (n=201)

Age, median (IQR)	60.8 (56.7–65.8)
Sex, n (%)—female	174 (86.6)
BMI, mean (SD)	26.7 (4.9)
Fulfilling ACR criteria for hand OA, n (%)	192 (95.5)
Fulfilling EULAR criteria for CMC1 OA only, n (%)	25 (12.4)
Fulfilling EULAR criteria for interphalangeal OA only, n (%)	95 (47.3)
Fulfilling EULAR criteria for both CMC1 and interphalangeal OA, n (%)	51 (25.4)
Dominant hand, n (%)—right hand*	186 (93.5)
Grip strength, dominant hand, mean (SD), kg	22.6 (9.1)
Symptom duration, median (IQR), years*	6 (3–13)
AUSCAN hand pain subscale (range 0–20), mean (SD)	8.1 (4.1)
AUSCAN hand function subscale (range 0–36), median (IQR)	11.3 (6–19)
Follow-up time, mean (SD), years	3.4 (0.4)

* Missing values: dominant hand n=2, symptom duration n=14.

ACR, American College of Rheumatology; AUSCAN, Australian/Canadian Osteoarthritis Hand Index; BMI, body mass index; CMC1, first carpometacarpal joint; EULAR, European Alliance of Associations for Rheumatology; OA, osteoarthritis.

**Table 2:** Baseline imaging characteristics of the study population (n=201)

Radiographic findings (joint level)	CMC1 joints	Interphalangeal joints
KL grade ≥2, n (%)*	215 (54.2)	1638 (45.4)
Osteophytes grade ≥1, n (%)*	252 (63.5)	1853 (51.4)
JSN grade ≥1, n (%)*	198 (49.9)	1512 (41.9)
Erosions (Verbruggen-Veys E or R phase), n (%)*	2 (0.5)	236 (6.5)
Ultrasonographic findings		
Grey-scale synovitis grade 0, n (%)*	295 (74.3)	2976 (82.6)
Grey-scale synovitis grade 1, n (%)*	40 (10.1)	400 (11.1)
Grey-scale synovitis grade 2, n (%)*	47 (11.8)	188 (5.2)
Grey-scale synovitis grade 3, n (%)*	15 (3.8)	38 (1.1)
Power Doppler activity grade 0, n (%)*	318 (80.1)	3259 (90.5)
Power Doppler activity grade 1, n (%)*	53 (13.4)	212 (5.9)
Power Doppler activity grade 2, n (%)*	23 (5.8)	117 (3.2)
Power Doppler activity grade 3, n (%)*	3 (0.8)	14 (0.4)
MRI findings (unilateral hand)		
Synovitis grade 0, n (%)*	54 (32.3)	698 (45.9)
Synovitis grade 1, n (%)*	78 (46.7)	624 (41.0)
Synovitis grade 2, n (%)*	27 (16.2)	153 (10.1)
Synovitis grade 3, n (%)*	8 (4.8)	46 (3.0)

* Missing values: KL grade CMC1 joints n=5, KL grade interphalangeal joints n=10, osteophytes grade CMC1 joints n=5, osteophytes grade interphalangeal joints n=10, JSN grade CMC1 joints n=5, JSN grade interphalangeal joints n=10, erosions CMC1 joints n=5, erosions interphalangeal joints n=11, grey-scale synovitis grade CMC1 joints n=5, grey-scale synovitis grade interphalangeal joints n=16, power Doppler activity grade CMC1 joints n=5, power Doppler activity grade interphalangeal joints n=16, MRI synovitis grade CMC1 joints n=34, MRI synovitis grade interphalangeal joints n=288.

CMC1, first carpometacarpal joint; JSN, joint space narrowing; KL, Kellgren-Lawrence.

The median age at baseline was 60.8 years (IQR 56.7–65.8) and 86.6% were women. The EULAR classification criteria for CMC1 OA and/or interphalangeal OA were fulfilled by 171 (85.1%) of the participants. Median symptom duration was 6 years (IQR 3–13). The mean follow-up time was 3.4 years (SD 0.4).

Reliability assessment showed weighted kappa values of 0.88 for CMC1 joints and 0.92 for interphalangeal joints for KL grade, 0.92 and 0.88 for osteophytes, 0.83 and 0.95 for JSN and kappa values of 1.00 and 0.98 for erosions, respectively.

Radiographic abnormalities were frequent. Definite OA, defined as KL grade ≥2 at baseline, was present in 54.2% and 45.4% of the CMC1 and interphalangeal joints, respectively. Erosions were rare in the CMC1 joints (0.5%), but present in 6.5% of the interphalangeal joints at baseline. Of joints with potential for radiographic KL progression, there was an increase in KL grade ≥1 in 14.7% of the CMC1 and 10.2% of the interphalangeal joints.

Ultrasound-detected grey-scale synovitis was found in 25.7% of the CMC1 joints and 17.4% of the interphalangeal joints, whereas power Doppler activity was present in 19.9% and 9.5%, respectively. MRI-detected synovitis was highly prevalent and observed in 67.7% of the CMC1 joints and 54.1% of the interphalangeal joints, of which the majority was grade 1 (table 2).

At the joint level, missing imaging data were limited except for MRI with 32 (15.9%) missing participants (tables 1 and 2 footnote). Joints with end-stage structural grades at baseline were excluded from the respective progression analyses, that is, joints with KL grade 4 (n=39 CMC1 joints and 286 interphalangeal joints), osteophyte grade 3 (n=32 CMC1 joints and 119 interphalangeal joints), JSN grade 3 (n=17 CMC1 joints and 277 interphalangeal joints) or erosive disease at baseline (n=2 CMC1 joints and 236 interphalangeal joints).

### Ultrasound synovitis as predictor for radiographic progression

A clear association between grey-scale synovitis and radiographic progression was observed in both the CMC1 and interphalangeal joints (table 3). In the interphalangeal joints, baseline synovitis was associated with both overall radiographic progression according to KL scores and progression of individual radiographic features, including osteophytes and JSN. In the CMC1 joints, all grades of grey-scale synovitis were associated with radiographic progression according to KL scores. There was a similar trend for osteophyte progression when synovitis was moderate to severe at baseline, whereas associations with JSN progression did not reach statistical significance. A strong association was observed for erosive development in the interphalangeal joints, whereas no development of erosions was observed in the CMC1 joints.

**Table 3:** Ultrasound grey-scale synovitis as predictor for radiographic OA progression in CMC1 and interphalangeal joints

CMC1 joints			Interphalangeal joints		
Baseline ultrasound	Radiographic progression, n (%)	Adjusted OR (95% CI)*	Baseline ultrasound	Radiographic progression, n (%)	Adjusted OR (95% CI)*
*Grey-scale synovitis*	*Kellgren-Lawrence grade*		*Grey-scale synovitis*	*Kellgren-Lawrence grade*	
Grade 0 (n=273)	28 (10.3)	1.0 (ref)	Grade 0 (n=2826)	206 (7.3)	1.0 (ref)
Grade 1 (n=32)	9 (28.1)	**2.8 (1.1 to 6.9)**	Grade 1 (n=323)	52 (16.1)	**2.2 (1.6 to 3.0)**
Grade 2 (n=37)	11 (29.7)	**4.0 (1.7 to 9.2)**	Grade 2 (n=140)	59 (42.1)	**7.8 (5.3 to 11.4)**
Grade 3 (n=6)	3 (50.0)	**9.3 (2.2 to 39.6)**	Grade 3 (n=29)	20 (69.0)	**23.4 (10.4 to 52.9)**
	*Osteophytes*			*Osteophytes*	
Grade 0 (n=276)	19 (6.9)	1.0 (ref)	Grade 0 (n=2909)	170 (5.8)	1.0 (ref)
Grade 1 (n=33)	4 (12.1)	1.4 (0.4 to 4.6)	Grade 1 (n=370)	63 (17.0)	**3.1 (2.2 to 4.4)**
Grade 2 (n=37)	10 (27.0)	**5.3 (2.2 to 12.7)**	Grade 2 (n=166)	66 (39.8)	**9.4 (6.5 to 13.6)**
Grade 3 (n=9)	2 (22.2)	**7.6 (1.4 to 39.6)**	Grade 3 (n=38)	21 (55.3)	**17.3 (8.4 to 35.6)**
	*Joint space narrowing*			*Joint space narrowing*	
Grade 0 (n=281)	20 (7.1)	1.0 (ref)	Grade 0 (n=2827)	105 (3.7)	1.0 (ref)
Grade 1 (n=36)	6 (16.7)	2.4 (0.7 to 7.8)	Grade 1 (n=327)	49 (15.0)	**3.9 (2.6 to 5.8)**
Grade 2 (n=44)	7 (15.9)	2.6 (0.7 to 8.9)	Grade 2 (n=144)	41 (28.5)	**8.2 (5.0 to 13.2)**
Grade 3 (n=9)	2 (22.2)	4.1 (0.7 to 22.2)	Grade 3 (n=29)	18 (62.1)	**32.1 (12.4 to 83.3)**
	*Erosions*			*Erosions*	
Grade 0 (n=287)	0 (0)	1.0 (ref)	Grade 0 (n=2843)	32 (1.1)	1.0 (ref)
Grade 1 (n=38)	0 (0)	na	Grade 1 (n=345)	32 (9.3)	**7.8 (4.5 to 13.2)**
Grade 2 (n=47)	0 (0)	na	Grade 2 (n=149)	28 (18.8)	**19.3 (10.9 to 33.9)**
Grade 3 (n=13)	0 (0)	na	Grade 3 (n=30)	12 (40.0)	**55.5 (23.2 to 132.7)**

Bold values indicate statistically significant associations (p<0.05).

* Adjusted for age, sex, body mass index and time between baseline and follow-up.

CMC1, first carpometacarpal joint; na, not applicable; OA, osteoarthritis; ref, reference.

Power Doppler activity was less frequent than grey-scale synovitis but showed a similar and consistent association with radiographic progression in both the CMC1 and interphalangeal joints (table 4). Increasing power Doppler activity grades were associated with progressively higher odds of KL progression in both joint groups, although with broader CIs for grade 2–3 power Doppler activity in CMC1 due to fewer joints included in these analyses.

**Table 4:** Ultrasound power Doppler activity as predictor for radiographic OA progression in CMC1 and interphalangeal joints

CMC1 joints			Interphalangeal joints		
Baseline ultrasound	Radiographic progression, n (%)	Adjusted OR (95% CI)*	Baseline ultrasound	Radiographic progression, n (%)	Adjusted OR (95% CI)*
*Power Doppler activity*	*Kellgren-Lawrence grade*		*Power Doppler activity*	*Kellgren-Lawrence grade*	
Grade 0 (n=294)	33 (11.2)	1.0 (ref)	Grade 0 (n=3064)	257 (8.4)	1.0 (ref)
Grade 1 (n=35)	10 (28.6)	**3.2 (1.3 to 7.7)**	Grade 1 (n=161)	49 (30.4)	**4.1 (2.8 to 5.9)**
Grade 2 (n=17)	7 (41.2)	**5.0 (1.7 to 14.5)**	Grade 2 (n=89)	28 (31.5)	**4.6 (2.9 to 7.4)**
Grade 3 (n=2)	1 (50.0)	10.7 (0.2 to 461.7)	Grade 3 (n=4)	3 (75.0)	**16.2 (1.6 to 164.5)**
	*Osteophytes*			*Osteophytes*	
Grade 0 (n=297)	22 (7.4)	1.0 (ref)	Grade 0 (n=3176)	225 (7.1)	1.0 (ref)
Grade 1 (n=38)	6 (15.8)	2.2 (0.9 to 5.8)	Grade 1 (n=190)	60 (31.6)	**5.5 (3.9 to 7.6)**
Grade 2 (n=18)	6 (33.3)	**5.0 (1.4 to 17.6)**	Grade 2 (n=108)	29 (26.9)	**4.4 (2.7 to 7.4)**
Grade 3 (n=2)	1 (50.0)	21.0 (0.2 to 1959.3)	Grade 3 (n=9)	6 (66.7)	**17.7 (5.0 to 63.0)**
	*Joint space narrowing*			*Joint space narrowing*	
Grade 0 (n=302)	21 (7.0)	1.0 (ref)	Grade 0 (n=3070)	147 (4.8)	1.0 (ref)
Grade 1 (n=45)	10 (22.2)	**4.2 (1.6 to 10.7)**	Grade 1 (n=162)	44 (27.2)	**6.1 (4.1 to 9.0)**
Grade 2 (n=20)	4 (20.0)	4.2 (0.8 to 20.9)	Grade 2 (n=89)	20 (22.5)	**4.9 (3.1 to 7.9)**
Grade 3 (n=3)	0 (0)	na	Grade 3 (n=6)	2 (33.3)	5.2 (0.2 to 140.8)
	*Erosions*			*Erosions*	
Grade 0 (n=308)	0 (0)	1.0 (ref)	Grade 0 (n=3095)	56 (1.8)	1.0 (ref)
Grade 1 (n=51)	0 (0)	na	Grade 1 (n=169)	31 (18.3)	**10.0 (5.5 to 18.2)**
Grade 2 (n=23)	0 (0)	na	Grade 2 (n=94)	13 (13.8)	**8.0 (4.1 to 15.8)**
Grade 3 (n=3)	0 (0)	na	Grade 3 (n=9)	4 (44.4)	**24.7 (2.4 to 250.7)**

Bold values indicate statistically significant associations (p<0.05).

* Adjusted for age, sex, body mass index and time between baseline and follow-up.

CMC1, first carpometacarpal joint; na, not applicable; OA, osteoarthritis; ref, reference.

### MRI-detected synovitis as predictor for radiographic progression

Baseline MRI-detected synovitis was associated with radiographic progression in both CMC1 and interphalangeal joints (table 5). Moderate and severe MRI synovitis (grades 2–3) showed strong associations with progression according to KL, osteophytes and JSN in both joint groups, whereas MRI synovitis grade 1 showed weaker and less consistent associations and did not reach statistical significance for all outcomes. MRI-detected synovitis was also associated with erosive development in interphalangeal joints with increasing risk across synovitis grades.

**Table 5:** MRI synovitis as predictor for radiographic OA progression in CMC1 and interphalangeal joints

CMC1 joints			Interphalangeal joints		
Baseline MRI	Radiographic progression, n (%)	Adjusted OR (95% CI)*	Baseline MRI	Radiographic progression, n (%)	Adjusted OR (95% CI)*
*MRI synovitis*	*Kellgren-Lawrence grade* †		*MRI synovitis*	*Kellgren-Lawrence grade*	
Grade 0 (n=51)	2 (3.9)	1.0 (ref)	Grade 0 (n=681)	40 (5.9)	1.0 (ref)
Grade 1 (n=73)	8 (11.0)	3.0 (0.6 to 15.0)	Grade 1 (n=586)	50 (8.5)	1.6 (1.0 to 2.6)
Grade 2 (n=17)	5 (29.4)	**10.3 (1.8 to 59.6)**	Grade 2 (n=122)	47 (38.5)	**11.9 (6.8 to 21.0)**
Grade 3 (n=5)	4 (80.0)	**100.8 (6.9 to 1464.4)**	Grade 3 (n=26)	20 (76.9)	**59.2 (20.9 to 168.2)**
	*Osteophytes*†			*Osteophytes*	
Grade 0 (n=51)	2 (3.9)	1.0 (ref)	Grade 0 (n=693)	26 (3.8)	1.0 (ref)
Grade 1 (n=73)	6 (8.2)	2.1 (0.4 to 11.2)	Grade 1 (n=600)	42 (7.0)	**2.2 (1.2 to 3.8)**
Grade 2 (n=18)	4 (22.2)	**7.0 (1.1 to 42.6)**	Grade 2 (n=136)	52 (38.2)	**20.0 (10.7 to 37.6)**
Grade 3 (n=6)	3 (50.0)	**23.1 (2.6 to 206.4)**	Grade 3 (n=40)	28 (70.0)	**72.1 (27.8 to 186.8)**
	*Joint space narrowing*			*Joint space narrowing*	
Grade 0 (n=53)	1 (1.9)	1.0 (ref)	Grade 0 (n=681)	13 (1.9)	1.0 (ref)
Grade 1 (n=75)	3 (4.0)	1.8 (0.2 to 19.3)	Grade 1 (n=587)	27 (4.6)	**2.5 (1.2 to 5.0)**
Grade 2 (n=23)	5 (21.7)	**14.6 (1.5 to 140.6)**	Grade 2 (n=125)	34 (27.2)	**20.3 (10.4 to 39.9)**
Grade 3 (n=6)	2 (33.3)	**20.3 (1.1 to 383.6)**	Grade 3 (n=27)	14 (51.9)	**61.5 (22.9 to 164.6)**
	*Erosions*			*Erosions*	
Grade 0 (n=54)	0 (0)	1.0 (ref)	Grade 0 (n=685)	3 (0.4)	1.0 (ref)
Grade 1 (n=76)	0 (0)	na	Grade 1 (n=590)	12 (2.0)	**4.6 (1.1 to 18.7)**
Grade 2 (n=25)	0 (0)	na	Grade 2 (n=128)	20 (15.6)	**45.4 (9.9 to 207.2)**
Grade 3 (n=7)	0 (0)	na	Grade 3 (n=29)	12 (41.4)	**228.2 (38.0 to 1368.7)**

Bold values indicate statistically significant associations (p<0.05).

* Adjusted for age, sex, body mass index and time between baseline and follow-up.

† Not adjusted for sex because no men experienced progression.

CMC1, first carpometacarpal joint; na, not applicable; OA, osteoarthritis; ref, reference.

Across all analyses (for both ultrasound and MRI), crude estimates were similar to the adjusted estimates, indicating that adjustment for age, sex, BMI and time between baseline and follow-up had minimal impact on the estimates (online supplemental tables 1–3).

## Discussion

In light of the 2023 EULAR classification criteria for hand OA, which distinguish CMC1 OA and interphalangeal OA as separate phenotypes,^12^ the present findings provide new insight into structural disease progression in hand OA. To our knowledge, the association between synovitis and subsequent radiographic progression has not previously been evaluated in CMC1 and interphalangeal joints separately in the same study. In the present study, radiographic progression occurred with comparable frequency in CMC1 and interphalangeal joints, and synovitis was consistently associated with progression in both joint groups. The exception was incident erosions, which only occurred in the interphalangeal joints.

Our findings are consistent with previous longitudinal studies demonstrating that imaging-detected synovitis predicts radiographic progression in hand OA. Mathiessen *et al* and Kortekaas *et al* showed that ultrasound-detected synovitis was associated with subsequent radiographic progression, while Haugen *et al* and Damman *et al* demonstrated similar prognostic associations using MRI.^7–9 11^ However, these studies analysed interphalangeal joints or hand OA as a single entity. The present study extends this literature by demonstrating that synovitis also has prognostic significance in the CMC1 joints when analysed as a distinct joint group.

Clinical manifestations of hand OA, including pain and functional impairment, may differ between CMC1 and interphalangeal joints, underscoring the relevance of analysing these joint groups separately. The CMC1 joint is exposed to substantial biomechanical load, has a unique saddle-shaped anatomy and relatively thick articular cartilage and plays a central role in pinch and grip function.^25^ In contrast to the CMC1 joint, the interphalangeal joints are hinge joints primarily exposed to repetitive flexion-extension movements, have thinner articular cartilage and lower multidirectional loads. These biomechanical and structural characteristics may modify the relationship between synovial inflammation and subsequent structural damage.^2 26^ Despite these differences, our findings indicate that inflammatory activity is relevant for radiographic progression in both joint groups.

With respect to imaging modality, findings across ultrasound and MRI consistently support inflammation as a prognostic marker, while also highlighting that modality and grading thresholds influence interpretation. Ultrasound provides direct assessment of synovial hypertrophy and vascularity, whereas MRI captures synovitis with high sensitivity, but may identify low-grade changes of uncertain prognostic relevance.^7–9^ In the present study, MRI detected low-grade synovitis (grade 1) more often than ultrasound. MRI-detected synovitis grade 1 did not consistently predict radiographic progression, similar to findings in another hand OA cohort.^8^ This may indicate that MRI grade 1 synovitis represents borderline inflammatory changes of uncertain clinical relevance, potentially also influenced by measurement variability. In the CMC1 joints, this may partly reflect limited statistical power, whereas this explanation is less applicable to analyses of the interphalangeal joints. In the Hand OSTeoArthritis in Secondary care cohort using TOMS, low-grade synovitis was more frequent than higher grades.^27^ In the subsequent full publication, analyses focused on MRI synovitis grade ≥2, reflecting uncertainty regarding the clinical relevance of low-grade synovitis findings.^28^ Together, these studies support our observation that higher grades of MRI-detected inflammation are more consistently associated with structural progression than grade 1 synovitis.

Although synovial inflammation seems to be associated with structural progression in hand OA, our results do not establish causality. Randomised controlled trials are therefore needed to determine whether targeting synovitis can modify structural disease progression (and potentially pain). The effect of intra-articular corticosteroid injections varies across joint groups. While a randomised controlled trial in interphalangeal OA demonstrated reduced pain during movement,^29^ randomised trials in CMC1 OA found no pain benefit compared with intra-articular saline or local anaesthesia,^30–32^ suggesting that inflammation may be less relevant in CMC1 OA. However, the present results, together with previous results from the Nor-Hand study,^13 14^ demonstrate that synovitis in the CMC1 joint is associated with both pain and structural progression.^10^ The conflicting results may reflect limitations of prior intervention studies, such as not including inflammation in the CMC1 joint as an inclusion criterion and small sample sizes in the previous studies published in full-text,^30 31^ rather than absence of an inflammatory contribution in CMC1 OA. Ongoing trials such as the Painful Inflammatory Carpometacarpal-1 osteoArthritis treated with intraarticular Steroids, Saline or an Occupational therapy intervention (PICASSO) trial are designed to address the effect of corticosteroid injection in the CMC1 joints, and may provide important insight into the potential of anti-inflammatory strategies to alter the course of CMC1 OA.^33^ Oral prednisolone has also improved both finger joint pain and thumb base pain compared with placebo after 6 weeks of treatment in patients with hand OA, supporting that synovitis may be an important cause of pain in both joint groups.^34^

Some limitations should be acknowledged. First, no formal sample size calculation was performed for the present analyses, and the smaller number of CMC1 joints may have reduced statistical power for some outcomes and contributed to imprecise estimates, particularly for severe synovitis grades, which were relatively uncommon. Nevertheless, several associations in CMC1 joints reached statistical significance and were consistent in direction with those observed in interphalangeal joints. Findings with few joints or events should therefore be interpreted with caution, while the overall pattern supports synovitis as a prognostic marker in both joint groups. Another potential weakness is that the participants were recruited from secondary care only, which may limit generalisability to individuals with hand OA managed in primary care. Strengths of this study include the large number of joints assessed, which enables robust evaluation of imaging findings in relation to radiographic progression, as well as the use of both ultrasound and MRI with good reliability.

In conclusion, baseline synovitis detected by ultrasound and MRI was associated with radiographic progression in both CMC1 and interphalangeal joints. These findings support inflammation as a relevant mechanism across hand OA phenotypes.

## Supplementary material

10.1136/rmdopen-2026-007074 online supplemental file 1

## Data Availability

Data are available on reasonable request.
