# Supplementary material for: Associations between synovitis and radiographic osteoarthritis progression in the first carpometacarpal and interphalangeal joints: results from the Nor-Hand study
Source: RMD Open. 2026 Aug 20;12(3):e007074. doi: 10.1136/rmdopen-2026-007074 (PMC13504491; doi:10.1136/rmdopen-2026-007074)
Supplement: Supplementary file 1 [file rmdopen-12-3-s001.docx]

**Suppl. Table 1 – Ultrasound grey-scale synovitis as predictor for radiographic OA progression in CMC1 and interphalangeal joints, presented as crude odds ratios**

| **CMC1 joints** |  |  | **Interphalangeal joints** |  |  |
| --- | --- | --- | --- | --- | --- |
| **Baseline ultrasound** | **Radiographic progression,**  **n (%)** | **Crude OR**  **(95% Cl)** | **Baseline ultrasound** | **Radiographic progression,**  **n (%)** | **Crude OR**  **(95% Cl)** |
| *Grey-scale synovitis* | *Kellgren-Lawrence grade* |  | *Grey-scale synovitis* | *Kellgren-Lawrence grade* |  |
| Grade 0 (n=273) | 28 (10.3) | 1.0 (ref) | Grade 0 (n=2826) | 206 (7.3) | 1.0 (ref) |
| Grade 1 (n=32) | 9 (28.1) | **3.1 (1.2 to 7.7)** | Grade 1 (n=323) | 52 (16.1) | **2.2 (1.6 to 3.1)** |
| Grade 2 (n=37) | 11 (29.7) | **3.7 (1.7 to 8.3)** | Grade 2 (n=140) | 59 (42.1) | **7.9 (5.4 to 11.5)** |
| Grade 3 (n=6) | 3 (50.0) | **7.5 (1.8 to 31.8)** | Grade 3 (n=29) | 20 (69.0) | **24.1 (10.7 to 54.5)** |
|  | *Osteophytes* |  |  | *Osteophytes* |  |
| Grade 0 (n=276) | 19 (6.9) | 1.0 (ref) | Grade 0 (n=2909) | 170 (5.8) | 1.0 (ref) |
| Grade 1 (n=33) | 4 (12.1) | 1.5 (0.5 to 5.2) | Grade 1 (n=370) | 63 (17.0) | **3.2 (2.3 to 4.4)** |
| Grade 2 (n=37) | 10 (27.0) | **4.5 (2.1 to 9.8)** | Grade 2 (n=166) | 66 (39.8) | **9.6 (6.6 to 13.8)** |
| Grade 3 (n=9) | 2 (22.2) | 4.6 (1.0 to 22.3) | Grade 3 (n=38) | 21 (55.3) | **17.9 (8.6 to 37.2)** |
|  | *Joint space narrowing* |  |  | *Joint space narrowing* |  |
| Grade 0 (n=281) | 20 (7.1) | 1.0 (ref) | Grade 0 (n=2827) | 105 (3.7) | 1.0 (ref) |
| Grade 1 (n=36) | 6 (16.7) | 2.5 (0.8 to 8.4) | Grade 1 (n=327) | 49 (15.0) | **3.9 (2.7 to 5.9)** |
| Grade 2 (n=44) | 7 (15.9) | 2.6 (0.8 to 8.5) | Grade 2 (n=144) | 41 (28.5) | **8.2 (5.2 to 13.1)** |
| Grade 3 (n=9) | 2 (22.2) | 3.6 (0.7 to 18.6) | Grade 3 (n=29) | 18 (62.1) | **34.0 (13.1 to 88.0)** |
|  | *Erosions* |  |  | *Erosions* |  |
| Grade 0 (n=287) | 0 (0) | 1.0 (ref) | Grade 0 (n=2843) | 32 (1.1) | 1.0 (ref) |
| Grade 1 (n=38) | 0 (0) | na | Grade 1 (n=345) | 32 (9.3) | **7.7 (4.6 to 12.9)** |
| Grade 2 (n=47) | 0 (0) | na | Grade 2 (n=149) | 28 (18.8) | **18.0 (10.6 to 30.6)** |
| Grade 3 (n=13) | 0 (0) | na | Grade 3 (n=30) | 12 (40.0) | **55.6 (23.8 to 129.8)** |

Cl = confidence interval, CMC1 = first carpometacarpal joint, OR = odds ratio. Na = not applicable

**Suppl. Table 2 – Ultrasound power Doppler activity as predictor for radiographic OA progression in CMC1 and interphalangeal joints, presented as crude odds ratios**

| **CMC1 joints** |  |  | **Interphalangeal joints** |  |  |
| --- | --- | --- | --- | --- | --- |
| **Baseline ultrasound** | **Radiographic progression,**  **n (%)** | **Crude OR**  **(95% Cl)** | **Baseline ultrasound** | **Radiographic progression,**  **n (%)** | **Crude OR**  **(95% Cl)** |
| *Power Doppler activity* | *Kellgren-Lawrence grade* |  | *Power Doppler activity* | *Kellgren-Lawrence grade* |  |
| Grade 0 (n=294) | 33 (11.2) | 1.0 (ref) | Grade 0 (n=3064) | 257 (8.4) | 1.0 (ref) |
| Grade 1 (n=35) | 10 (28.6) | **3.4 (1.4 to 8.0)** | Grade 1 (n=161) | 49 (30.4) | **4.2 (2.9 to 5.9)** |
| Grade 2 (n=17) | 7 (41.2) | **5.2 (2.0 to 13.4)** | Grade 2 (n=89) | 28 (31.5) | **4.7 (2.9 to 7.7)** |
| Grade 3 (n=2) | 1 (50.0) | 6.6 (0.2 to 243.0) | Grade 3 (n=4) | 3 (75.0) | **18.3 (1.7 to 200.4)** |
|  | *Osteophytes* |  |  | *Osteophytes* |  |
| Grade 0 (n=297) | 22 (7.4) | 1.0 (ref) | Grade 0 (n=3176) | 225 (7.1) | 1.0 (ref) |
| Grade 1 (n=38) | 6 (15.8) | 2.2 (0.9 to 5.4) | Grade 1 (n=190) | 60 (31.6) | **5.5 (4.0 to 7.7)** |
| Grade 2 (n=18) | 6 (33.3) | **5.1 (1.6 to 16.2)** | Grade 2 (n=108) | 29 (26.9) | **4.6 (2.7 to 7.8)** |
| Grade 3 (n=2) | 1 (50.0) | 8.8 (0.2 to 336.0) | Grade 3 (n=9) | 6 (66.7) | **18.6 (5.1 to 68.6)** |
|  | *Joint space narrowing* |  |  | *Joint space narrowing* |  |
| Grade 0 (n=302) | 21 (7.0) | 1.0 (ref) | Grade 0 (n=3070) | 147 (4.8) | 1.0 (ref) |
| Grade 1 (n=45) | 10 (22.2) | **4.4 (1.8 to 10.8)** | Grade 1 (n=162) | 44 (27.2) | **6.2 (4.2 to 9.1)** |
| Grade 2 (n=20) | 4 (20.0) | 4.4 (0.9 to 22.1) | Grade 2 (n=89) | 20 (22.5) | **5.1 (3.2 to 8.2)** |
| Grade 3 (n=3) | 0 (0) | na | Grade 3 (n=6) | 2 (33.3) | 5.5 (0.1 to 220.9) |
|  | *Erosions* |  |  | *Erosions* |  |
| Grade 0 (n=308) | 0 (0) | 1.0 (ref) | Grade 0 (n=3095) | 56 (1.8) | 1.0 (ref) |
| Grade 1 (n=51) | 0 (0) | na | Grade 1 (n=169) | 31 (18.3) | **10.1 (5.6 to 18.1)** |
| Grade 2 (n=23) | 0 (0) | na | Grade 2 (n=94) | 13 (13.8) | **8.2 (4.1 to 16.4)** |
| Grade 3 (n=3) | 0 (0) | na | Grade 3 (n=9) | 4 (44.4) | **25.8 (2.6 to 258.6)** |

Cl = confidence interval, CMC1 = first carpometacarpal joint, OR = odds ratio. Na = not applicable

**Suppl. Table 3 – MRI synovitis as predictor for radiographic OA progression in CMC1 and interphalangeal joints, presented as crude odds ratios**

| **CMC1 joints** |  |  | **Interphalangeal joints** |  |  |
| --- | --- | --- | --- | --- | --- |
| **Baseline MRI** | **Radiographic progression,**  **n (%)** | **Crude OR**  **(95% Cl)** | **Baseline MRI** | **Radiographic progression,**  **n (%)** | **Crude OR**  **(95% Cl)** |
| *MRI synovitis* | *Kellgren-Lawrence grade** |  | *MRI synovitis* | *Kellgren-Lawrence grade* |  |
| Grade 0 (n=51) | 2 (3.9) | 1.0 (ref) | Grade 0 (n=681) | 40 (5.9) | 1.0 (ref) |
| Grade 1 (n=73) | 8 (11.0) | 3.0 (0.6 to 14.8) | Grade 1 (n=586) | 50 (8.5) | 1.6 (1.0 to 2.6) |
| Grade 2 (n=17) | 5 (29.4) | **10.2 (1.8 to 59.2)** | Grade 2 (n=122) | 47 (38.5) | **11.0 (6.4 to 18.8)** |
| Grade 3 (n=5) | 4 (80.0) | **98.0 (7.2 to 1329.9)** | Grade 3 (n=26) | 20 (76.9) | **53.1 (18.8 to 149.6)** |
|  | *Osteophytes** |  |  | *Osteophytes* |  |
| Grade 0 (n=51) | 2 (3.9) | 1.0 (ref) | Grade 0 (n=693) | 26 (3.8) | 1.0 (ref) |
| Grade 1 (n=73) | 6 (8.2) | 2.2 (0.4 to 11.3) | Grade 1 (n=600) | 42 (7.0) | **2.1 (1.2 to 3.6)** |
| Grade 2 (n=18) | 4 (22.2) | **7.0 (1.2 to 42.3)** | Grade 2 (n=136) | 52 (38.2) | **17.7 (10.0 to 31.3)** |
| Grade 3 (n=6) | 3 (50.0) | **24.5 (2.9 to 207.3)** | Grade 3 (n=40) | 28 (70.0) | **61.3 (24.1 to 155.8)** |
|  | *Joint space narrowing* |  |  | *Joint space narrowing* |  |
| Grade 0 (n=53) | 1 (1.9) | 1.0 (ref) | Grade 0 (n=681) | 13 (1.9) | 1.0 (ref) |
| Grade 1 (n=75) | 3 (4.0) | 2.2 (0.2 to 21.4) | Grade 1 (n=587) | 27 (4.6) | **2.5 (1.2 to 5.0)** |
| Grade 2 (n=23) | 5 (21.7) | **14.4 (1.6 to 132.1)** | Grade 2 (n=125) | 34 (27.2) | **19.2 (9.9 to 37.2)** |
| Grade 3 (n=6) | 2 (33.3) | **26.0 (1.9 to 352.5)** | Grade 3 (n=27) | 14 (51.9) | **54.2 (19.9 to 147.6)** |
|  | *Erosions* |  |  | *Erosions* |  |
| Grade 0 (n=54) | 0 (0) | 1.0 (ref) | Grade 0 (n=685) | 3 (0.4) | 1.0 (ref) |
| Grade 1 (n=76) | 0 (0) | na | Grade 1 (n=590) | 12 (2.0) | **4.5 (1.2 to 17.8)** |
| Grade 2 (n=25) | 0 (0) | na | Grade 2 (n=128) | 20 (15.6) | **38.6 (9.7 to 154.3)** |
| Grade 3 (n=7) | 0 (0) | na | Grade 3 (n=29) | 12 (41.4) | **152.7 (30.2 to 771.6)** |

Cl = confidence interval, CMC1 = first carpometacarpal joint, MRI = magnetic resonance imaging, OR = odds ratio.

Na = not applicable.

*Not adjusted for sex due to no men with progression
